# Neural Networks for Mindfulness and Emotion Suppression

**DOI:** 10.1371/journal.pone.0128005

**Published:** 2015-06-17

**Authors:** Hiroki Murakami, Ruri Katsunuma, Kentaro Oba, Yuri Terasawa, Yuki Motomura, Kazuo Mishima, Yoshiya Moriguchi

**Affiliations:** 1 Integrated Neuroscience Research Project, Tokyo Metropolitan Institute of Medical Science, Tokyo, Japan; 2 Department of Psychophysiology, National Institute of Mental Health, National Center of Neurology and Psychiatry, Kodaira, Tokyo, Japan; 3 Integrative Brain Imaging Center, National Center of Neurology and Psychiatry, Kodaira, Tokyo, Japan; 4 Graduate School of Health Sciences, Gunma University, Maebashi, Gunma, Japan; Saybrook University, UNITED STATES

## Abstract

Mindfulness, an attentive non-judgmental focus on “here and now” experiences, has been incorporated into various cognitive behavioral therapy approaches and beneficial effects have been demonstrated. Recently, mindfulness has also been identified as a potentially effective emotion regulation strategy. On the other hand, emotion suppression, which refers to trying to avoid or escape from experiencing and being aware of one’s own emotions, has been identified as a potentially maladaptive strategy. Previous studies suggest that both strategies can decrease affective responses to emotional stimuli. They would, however, be expected to provide regulation through different top-down modulation systems. The present study was aimed at elucidating the different neural systems underlying emotion regulation via mindfulness and emotion suppression approaches. Twenty-one healthy participants used the two types of strategy in response to emotional visual stimuli while functional magnetic resonance imaging was conducted. Both strategies attenuated amygdala responses to emotional triggers, but the pathways to regulation differed across the two. A mindful approach appears to regulate amygdala functioning via functional connectivity from the medial prefrontal cortex, while suppression uses connectivity with other regions, including the dorsolateral prefrontal cortex. Thus, the two types of emotion regulation recruit different top-down modulation processes localized at prefrontal areas. These different pathways are discussed.

## Introduction

Emotion regulation is a fundamental function for human beings, as social animals, to adapt to the environment. There are a number of emotion regulation strategies, including emotion suppression, reappraisal [1], distraction [2], and detachment [3]. Recently, *mindfulness* has received focus as an effective emotion regulation strategy [4–6]. Mindfulness has been defined as “paying attention in a particular way: on purpose, in the present moment, and nonjudgmentally” (Kabat-Zinn, 1994, p. 4 [7]). More specifically, Baer et al. (2006) identified the central components of mindfulness, as described below. Mindfulness emphasizes the importance of observing a wide range of stimuli as a whole, including one’s own internal sensations as well as external phenomena outside the body. Moreover, mindfulness encourages describing observed phenomena, within the body and in the outer world, and engaging fully in one’s current activity with undivided attention, such that awareness is focused on one experience at a time, but not completely caught or involved in the experiences, i.e., focused attention with keeping some distance between oneself and the experiences. Additionally, mindfulness emphasizes accepting, allowing, or being nonjudgmental or nonevaluative about an experience in the present moment [8]. These core components of mindfulness have been introduced to various cognitive behavioral therapy approaches, including Mindfulness-Based Cognitive Therapy (MBCT) [9], Acceptance and Commitment Therapy (ACT) [10], and Dialectical Behavior Therapy (DBT) [11].

Beneficial effects of mindfulness training have been demonstrated in several studies. In a one-year follow-up study, mindfulness was shown to have significant efficacy in reducing relapse and recurrence of major depression, compared with treatment as usual [9]. Another one-year follow-up study with breast and prostate cancer patients showed that mindfulness reduced stress-related indices such as inflammatory cytokines and cortisol as well as stress symptoms per se [12]. Baer (2003) suggested the possibility that mindfulness skill practice improves patients’ ability to effectively tolerate and cope with negative emotional states, by extinguishing fear responses and avoidance behaviors previously elicited by these stimuli [13].

On the other hand, *emotion suppression* refers to attempts to avoid or escape from experiencing and being aware of one’s own emotions. Researchers have generally viewed emotion suppression as a maladaptive emotion regulation strategy [1], such that individuals who frequently suppress experience less positive emotion, with a corresponding increased likelihood of experiencing negative emotions that are negatively associated with diverse aspects of psychosocial wellness [14]. Additionally, suppression of negative emotion promotes exaggerated sympathetic nervous system activity that can affect immune responses and physical health [1]. Contrary to emotion suppression, mindfulness emphasizes an increasing awareness and full acceptance of all emotional experiences, regardless of apparent valence, intensity, or perceived utility [15]. It is quite difficult for human beings to suppress feelings, given that affective changes *per se* are inherent and spontaneous, such that the acceptance of one’s own emotions should help one to stop endless and inefficient efforts to avoid emotional experiences. In fact, mindfulness increases parasympathetic nervous system activity and in essence ‘calms down’ systemic arousal [16,17], and thereby promotes therapeutic effects on psychopathological disorders [9,12].

Understanding underlying mechanisms of mindfulness has been a hot topic in recent years, not only in clinical fields but also in neuroscience (see Tang & Posner, 2013 [18]). Previous neuroimaging studies of mindfulness suggested that mindful participants show greater activation in the medial prefrontal cortex (MPFC) and lower activation in the amygdala (AMG) in response to emotional stimuli [19,20] Considering that the AMG plays a primary role in emotional responses [21], these studies suggest that mindfulness improves emotion regulation via MPFC activity, which has an inhibitory effect on the AMG [22–24]. Electrical stimulation of the MPFC decreases the excitability of the projecting neurons from the central nucleus of the AMG to the brainstem, decreasing the expression of conditioned fear in rodents [25]. Similarly, MPFC functioning is related to extinction learning in human beings [26].

In contrast, neuroimaging studies have not provided a conclusive answer regarding whether emotion suppression is effective in regulating emotional responses. One study showed marginally elevated AMG responding during expressive suppression (voluntarily suppressing outward emotional expressions) [27]. However, another investigation showed that engaging in expressive suppression was effective in reducing AMG activity in response to emotional stimuli [28]. Ohira et al. (2006) also showed that voluntary emotion suppression while viewing an affective stimulus successfully attenuates AMG responses, while simultaneously measured physiological emotional responding (peripheral skin conductance) was actually enhanced during the suppression task [29].

Thus, evidence from neuroimaging studies suggests that both types of emotion regulation strategy–mindfulness and suppression–can possibly work to reduce AMG activity in response to emotional stimuli. However, the different strategies produce distinct patterns of peripheral emotional responses, such that mindfulness is associated with parasympathetic activity whereas suppression is correlated with sympathetic activity. This pattern could be due to processing differences between the two strategies, which would be expected to reflect different neural bases underlying the two strategies.

Contrary to the engagement of the MPFC during mindfulness, the lateral part of the prefrontal cortex typically contributes to the regulation of emotional responses during suppression [27,29,30]. The emotional suppression process localized at the lateral prefrontal cortex might nevertheless be mentally stressful to engage. For example, the dorsolateral prefrontal cortex (DLPFC) is activated during working memory tasks [31] that are frequently used to induce mental stress [32,33], and that such activation becomes more pronounced as task difficulty increases [34]. Such stressful cognitive processing may leave fewer cognitive resources available for concurrent emotional processing, which could work to control emotional responses even as cognitive stress may concurrently promote peripheral hyper-arousal. One plausible hypothesis here is that both mindfulness and suppression strategies can decrease AMG activity in response to emotional stimuli through different top-down modulation systems. No study, however, has directly compared the neural correlates of mindfulness and emotional suppression, which could provide further insight into the crucial components of emotion regulation.

The present study sought to elucidate neural differences between mindfulness and suppression emotion regulation strategies by way of a direct comparison between the two. We used functional connectivity analyses to identify different brain regions that contribute to regulating AMG responses during exposure to emotional stimuli, with the goal of identifying possible differences in the brain regions involved. AMG has been shown to have "negative" functional connectivity to the prefrontal cortex, and the MPFC in particular (e.g., [23,24, 26,35]), as well as lateral prefrontal areas (e.g., [23,36,37]). We expected that during mindfulness, the MPFC, which reportedly facilitates extinction learning, would be engaged to regulate AMG activation through negative functional coupling (i.e., negative correlation of time-series signals between the AMG and MPFC). On the other hand, during emotion suppression the lateral prefrontal cortex was expected to regulate AMG activation as a consequence of cognitive effort.

## Materials and Methods

### Participants

Twenty-one healthy, right-handed volunteers (10 men, 11 females, mean (±SD) age = 25.1 (±5.5) years, age range = 20–41 years) participated in the study. None of the participants reported a history of neurological or psychiatric disorders or current use of medication. All participants provided written informed consent in accordance with the Declaration of Helsinki. The study was approved by the Ethics Committee of the National Center of Neurology and Psychiatry, Japan.

### Materials

Twenty-one neutral and 63 negative pictures were selected from the International Affective Picture System (IAPS), a data set that has been carefully characterized with respect to both valence and arousal [38]. Based on normative ratings [38], negative pictures were divided into three sets for use in the three task conditions described below, matched on emotional valence and arousal. Mean (SD) valence scores (1 = negative, 5 = neutral, 9 = positive) for the neutral and the three sets of negative pictures were 5.00 (SD = .51), 2.94 (.84), 2.87 (.72), and 2.93 (.90), respectively, and the scores did not differ between the three sets of negative pictures, *F*(2,60) = .046, *p* = .955. Mean (SD) arousal scores were 2.58 (.34), 6.04 (.60), 6.04 (.77) and 6.02 (.70), respectively, and again three were no differences between the negative sets, *F*(2,60) = .004, *p* = .997. We had the participants rate the pictures prior to the fMRI study, confirming that the pictures had similar valence and arousal values for the participants in our study: Mean (SD) valence scores were 5.20 (.23), 3.11 (.53), 2.94 (.57), and 3.10 (.59), respectively, and the scores did not differ across the three sets of negative pictures *F*(3,20) = .629, *p* = .536, and the mean (SD) arousal scores were 2.56 (1.15), 6.27 (.90), 6.50 (.79) and 6.16 (.90), respectively, with no differences across the latter three sets, *F*(3,20) = .892, *p* = .415.

### Procedure

Blood oxygen level dependent (BOLD) signal changes were measured during four experimental tasks: Look-neutral, Look-negative, Suppress-negative, and Observe-negative. During Look-neutral and Look-negative tasks, the participants were required to simply look at the neutral or negative pictures and respond naturally. During Suppress-negative, the participants were required to voluntarily suppress any emotional responses to viewing negative pictures. Specifically, they were required to try to "remain calm and to diminish any subjective feelings regardless of the affective valence of the stimulus", which followed the procedure used by Ohira et al. (2006) [29]. During the Observe-negative task, a mindfulness intervention, participants were required to "observe objectively and describe their subjective feelings or thoughts in their minds, and physiological changes in bodies, not with voice but just mentally, and to *not* suppress the emotions that are evoked by viewing the negative pictures". Before entering the MRI scanner, participants were carefully instructed regarding how to cope with the presented affective pictures using the three strategies (i.e., Look, Suppress, and Observe).

The participants each took part in three fMRI runs, each of which consisted of 28 trials. Each trial was composed of four successive events as follows. First, a cue word (“Look”, “Suppress”, or “Observe”) appeared centrally on the screen for 2 s right before the presentation of an IAPS picture. Only neutral pictures were presented during the Look-neutral task, while negative pictures were presented during the other tasks. During the same period, picture valence ("neutral" or "negative") was shown below the cue word to avoid potential effects of uncertainty of picture valances across the task conditions [39]. Second, a neutral or negative picture appeared centrally for 8 s. While the picture remained on the screen, participants were required to perform the coping strategy/action specified by the prior cue. Third, a 9-point Likert scale (1 = none, 9 = extremely) appeared immediately after presentation of the picture. This scale allowed the participants to rate the current strength of subjective negative affect in their own mind after experiencing the picture stimulus while engaging in the indicated coping strategy. Participants provided these ratings by moving an MRI-compatible trackball. Fourth, a fixation cross appeared for 4 s in the center of the screen, indicating that participants should relax until the next trial began. In each run the order of the four task conditions (Look-neutral, Look-negative, Suppress-negative, and Observe-negative) was presented pseudorandom order in an event related design. We counterbalanced set of negative pictures used in each task condition across participants.

After fMRI scanning, we asked the participants whether they had successfully observed themselves objectively using the following question: “How much did you observe yourself objectively during the [Look, Suppress, or Observe] task in the scanner?” They rated themselves, separately for each task condition, using 9-point Likert scales (1 = not at all, 9 = very much so). We expected them to report greater levels of objective observation during the Observe task than during the other tasks. We dubbed this score “objective perspective” and used it to assess whether the Observe task successfully induced a mindful state.

### Image Acquisition and Analyses

Magnetic resonance images were acquired on a 1.5-T Siemens Magnetom Vision Plus System. To obtain a blood-oxygenation-level dependent (BOLD) contrast for functional imaging [40], changes in the T2*-weighted magnetic resonance (MR) signal were measured using a gradient echo-planar imaging (EPI) sequence (repetition time (TR) = 2500 ms, echo time (TE) = 40 ms, field of view (FOV) = 192 mm, flip angle = 90 degree, 64 × 64 matrix, 31 slices per slab, slice thickness 4 mm, 1 mm gap, voxel size = 3 × 3 × 4 mm). For each scanning run, a total of 236 EPI volume images were acquired along the AC-PC plane. The first five EPI image volumes were discarded because of magnetization instability, such that we obtained 231 EPI volumes for analyses.

Image processing and statistical analyses were carried out using Statistical Parametric Mapping software (SPM8, Wellcome Department, London, UK). Preprocessing of the functional scans included realignment for motion correction using the first scan as a reference and spatial normalization to a standard template (Montreal Neurological Institute, MNI), with a resampled voxel size of 2 × 2 × 2 mm. The normalized images were smoothed using an 8-mm FWHM Gaussian kernel. Intrinsic autocorrelations were accounted for by AR(1) and low frequency drifts were removed via high pass filter (128 s). The 2 s instruction period, the 8 s regulation period, and 6 s rating period were modeled as a delayed boxcar regressor convolved with the canonical hemodynamic response. The time-course imaging data were regressed using a linear combination of the differential hypothetical hemodynamic time-courses of different event types on a voxel-by-voxel basis. This general linear model (GLM) analysis produced contrast images for each participant which included the differential parameter estimates between task conditions, which were taken to a second-level random effects model for group analyses.

To identify emotion-related brain regions, we first sought significantly-activated clusters for Look-negative contrasted with Look-neutral (height threshold, *p* < .001 uncorrected, extent threshold, *k* = 5 voxels), and made a 3mm-sphere region of interest (ROI) centered on the peak of the cluster. We calculated individual mean contrast values for 1) Look-negative vs. Look-neutral, 2) Suppress-negative vs. Look-negative, and 3) Observe-negative vs. Look-negative within the ROI using Marsbar software (http://marsbar.sourceforge.net).

To identify brain regions remotely connected to emotional regions above, voxel-based and ROI-based correlational analyses were performed using the Functional Connectivity toolbox (CONN; http://www.nitrc.org/projects/conn/). After pre-processing of functional images, time-series data for each voxel were filtered with a band-pass of .01 – .20 Hz. This band-pass range had been optimized to capture effective hemodynamic changes by use of the spectral analyses of the aforementioned GLM model. The CONN toolbox was used to perform seed-based functional connectivity analysis, by computing Fisher-transformed bivariate temporal correlation coefficients between BOLD time series at a given ROI and those at all other voxels in the brain. The toolbox also extracts signals from the white matter (WM) and cerebrospinal fluid (CSF), which should reflect physiological fluctuation. Then, WM and CSF signals, together with motion parameters, were regressed out as nuisance variables during the functional connectivity analyses. We created a correlational brain map, each voxel of which contains the functional connectivity between that voxel and the seed ROI.

Finally, to identify differential functional connectivity that might produce differential emotional responses between the two strategies, we investigated the relationship between the magnitude of activation at the emotional ROI and the functional connectivity to that ROI. We calculated individual functional connectivity to the ROI during each Suppress-, Observe-, and Look-negative strategy, then contrasted the first two with the last (i.e., Suppress vs. Look, Observe vs. Look). And individual regional responses during Suppress and Observe (contrasted with Look) were also extracted from the ROI. These regional responses to Suppress and Observe were correlated with the differential functional connectivity (height threshold, *p* < .001 uncorrected, extent threshold, *k* = 5 voxels).

## Results

### Behavioral results

We first confirmed whether our experimental instructions for the Observe task were successful. A one-way ANOVA (Look / Suppress / Observe) conducted on the objective perspective scores showed a significant difference across the three tasks: Mean (and standard deviations) scores for the Look, Suppress, and Observe tasks were 2.29 (1.34), 3.05 (1.49), and 6.76 (1.26), respectively, *F* (2, 40) = 86.83, *p* = 1×10^-14^, *η* ^2^_*p*_ = .81. Specifically, objective perspective scores were significantly greater for the Observe task than for the other tasks (compared with Look, *T*(20) = 12.32, *p* = 7×10^-10^; with Suppress, *T*(20) = 10.22, *p* = 1×10^-8^, after Bonferroni correction), indicating that the participants felt that they could observe themselves objectively during the appropriate task.

**Fig 1:**
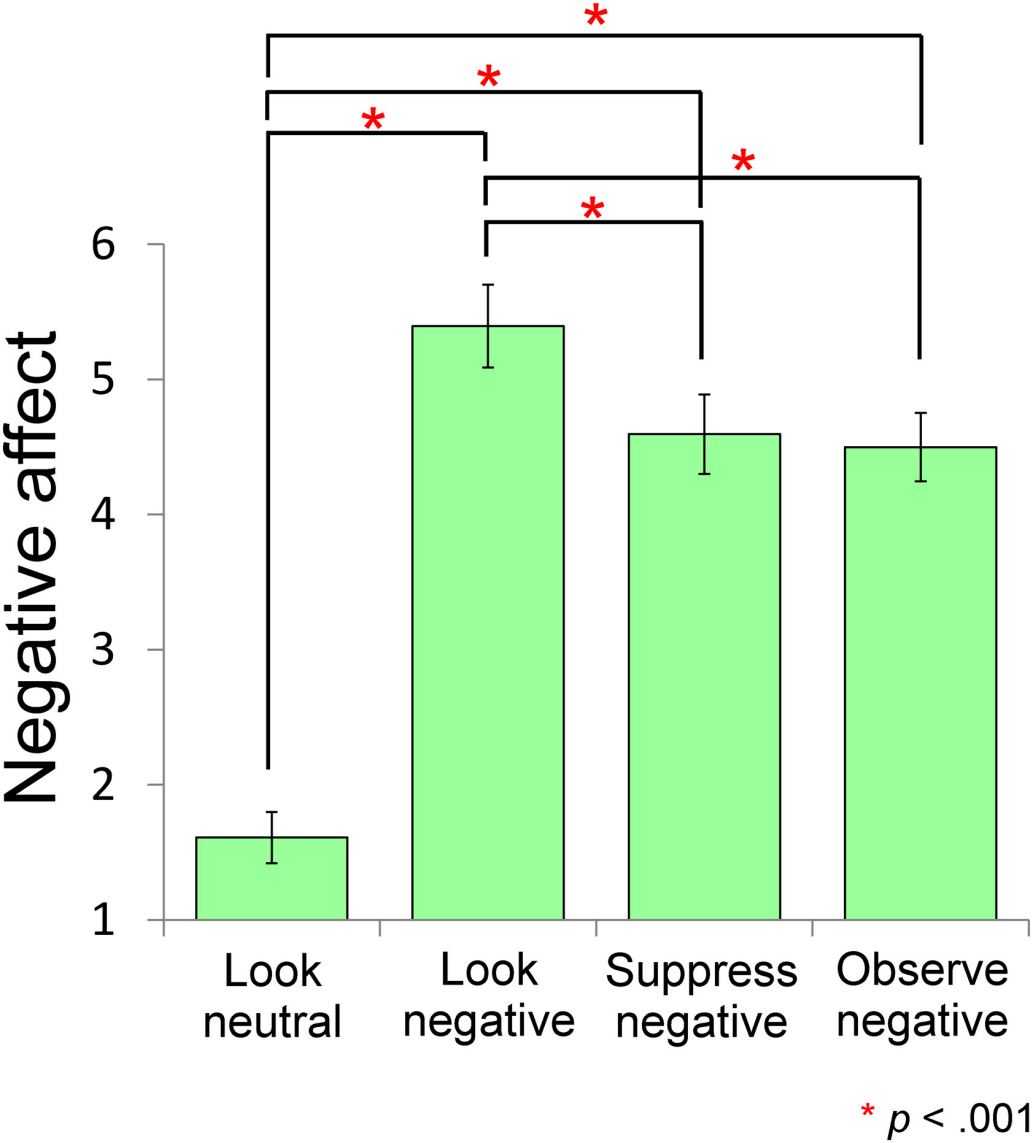
VAS scores for negative affect after each coping strategy (bars represent standard errors). Significant differences were found for the comparisons of Look-neutral vs. other conditions and Look-negative vs. Suppress-negative, and Observe-negative. The two types of emotion regulation strategies were effective for regulation of subjective emotion.

Participants’ negative affect scores were significantly different after engaging in the four tasks, *M* (*SD*) = 1.61 (.87), 5.39 (1.41), 4.59 (1.35) and 4.50 (1.16) for Look-neutral, Look-negative, Suppress-negative, and Observe-negative, respectively, *F* (3,60) = 95.11, *p* = 9×10^-23^, *η* ^2^_*p*_ = .89 (see Fig 1). We confirmed that presentation of the negative pictures enhanced negative emotionality in the participants: Negative affect scores after the Look-neutral task were significantly lower than scores for the other three tasks where negative pictures were presented (*t*(20) = 11.99, *p* = 1×10^-10^ for comparison with Look-negative, *t*(20) = 10.34, *p* = 2×10^-9^ for comparison with Suppress-negative, and *t*(20) = 12.08, *p* = 1×10^-10^ for comparison with Observe-negative, after Bonferroni correction). Importantly, both suppression and observation strategies were effective for regulation of emotion: Negative affect scores after Suppress-negative (*t*(20) = 3.33, *p* = .001, with Bonferroni correction) and Observe-negative (*t*(20) = 3.73, *p* = .001, with Bonferroni correction) task were lower than scores after the Look-negative task.

### fMRI results

#### Neural response in the AMG in different coping strategies

**Fig 2:**
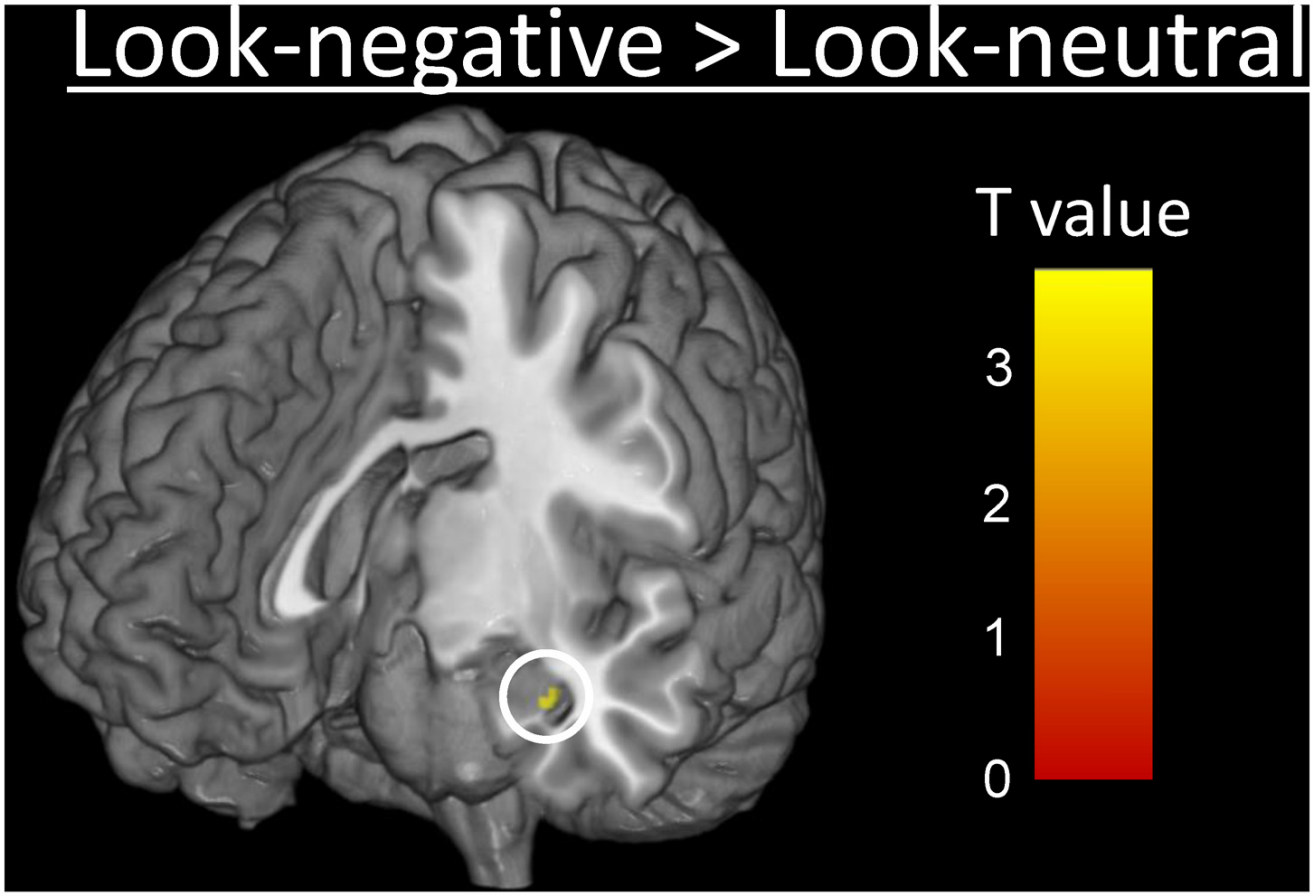
Left AMG [-34, -8, -22] response to affective stimuli (Look-negative vs. Look-neutral contrast (*p* < .001 uncorrected for illustration)).

The whole brain analysis for the contrast between Look-negative and Look-neutral showed activations in emotion-related brain areas such as the AMG and midbrain, as well as other regions such as the occipital gyrus, prefrontal cortex, and dorsal anterior cingulate cortex (dACC) (S1 Table). As expected, the left AMG showed a robust response to the affective stimuli (Look-negative vs. Look-neutral contrast; *t*(60) = 3.71, *p* = .0002 (uncorrected), cluster *k* = 9 voxels; peak MNI coordinate (mm) *x* = −34, *y* = −8, *z* = −22; Fig 2). The AMG has been frequently described as the center of emotional processing in the brain [21,25], such that we focused this region, creating a 3-mm sphere ROI centered on the peak coordinate.

**Fig 3:**
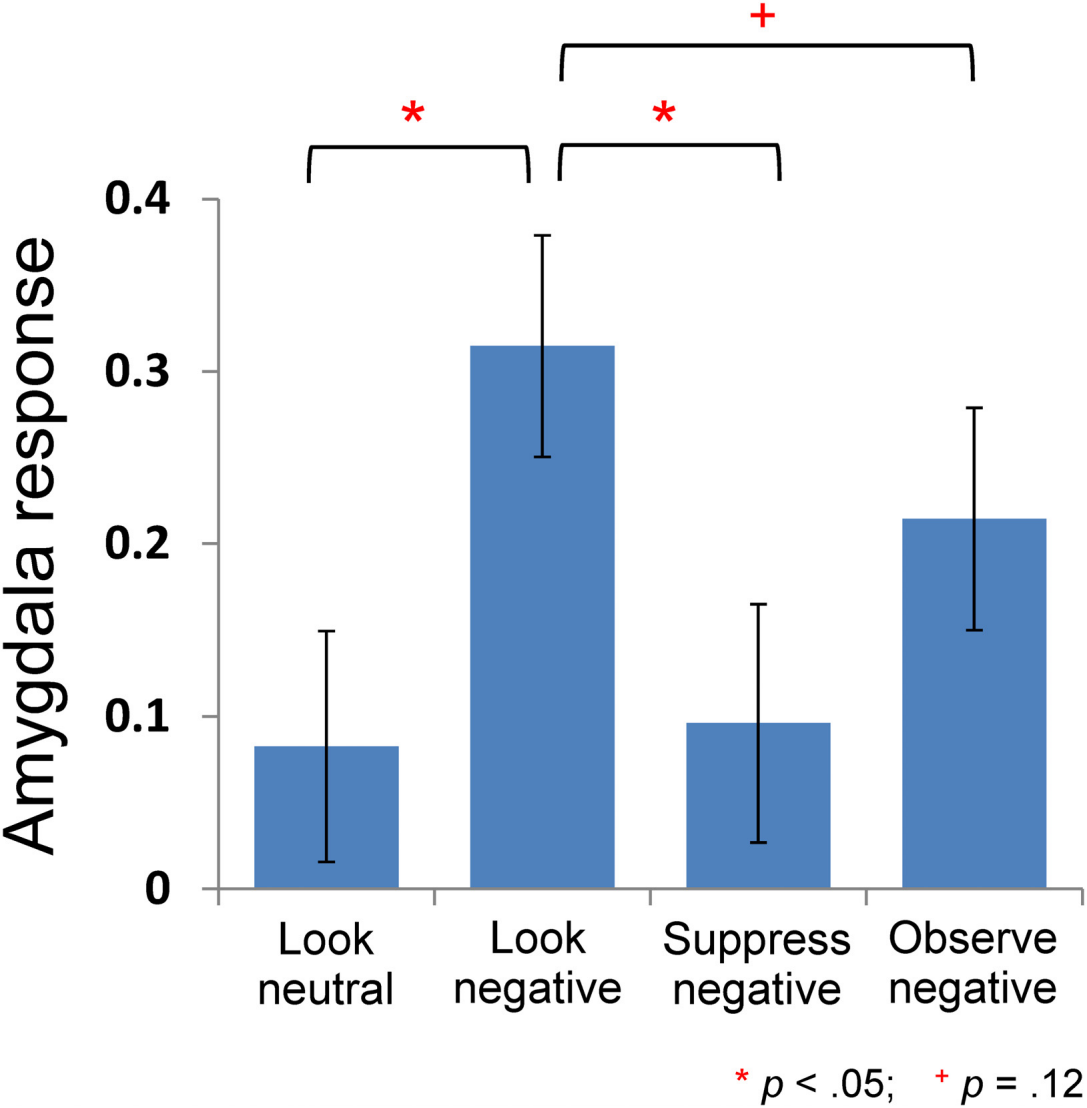
Neural response in the left AMG ROI in each condition (bars represent standard errors). The response to Look-negative was significantly stronger than the response to Look-neutral. The response to Suppress-negative and Observe-negative was significantly lower than the response to Look-negative, although the difference between Observe-negative and Look-negative was marginal.

AMG responses while viewing negative pictures (Look-negative, Suppress-negative, and Observe-negative) were significantly different from each other, *F* (2, 40) = 5.92, *p* = .009, *η* ^2^_*p*_ = .23 (Fig 3). More specifically, AMG responding during Suppress-negative was significantly lower than during Look-negative, *t*(20) = 3.44, *p* = .0014, such that suppression was effective to regulate emotional responding in the AMG. AMG responding during Observe-negative was marginally lower than responding during Look-negative, *t*(20) = 1.57, *p* = .12, which suggests that the observation strategy also worked to regulate emotional responding in the AMG although the effect was marginal, and marginally less robust than the effect of suppression *t*(20) = 2.51, *p* = .06 (Suppress-Negative vs. Observe-Negative).

#### Whole brain analysis for effect of emotion regulation

**Fig 4:**
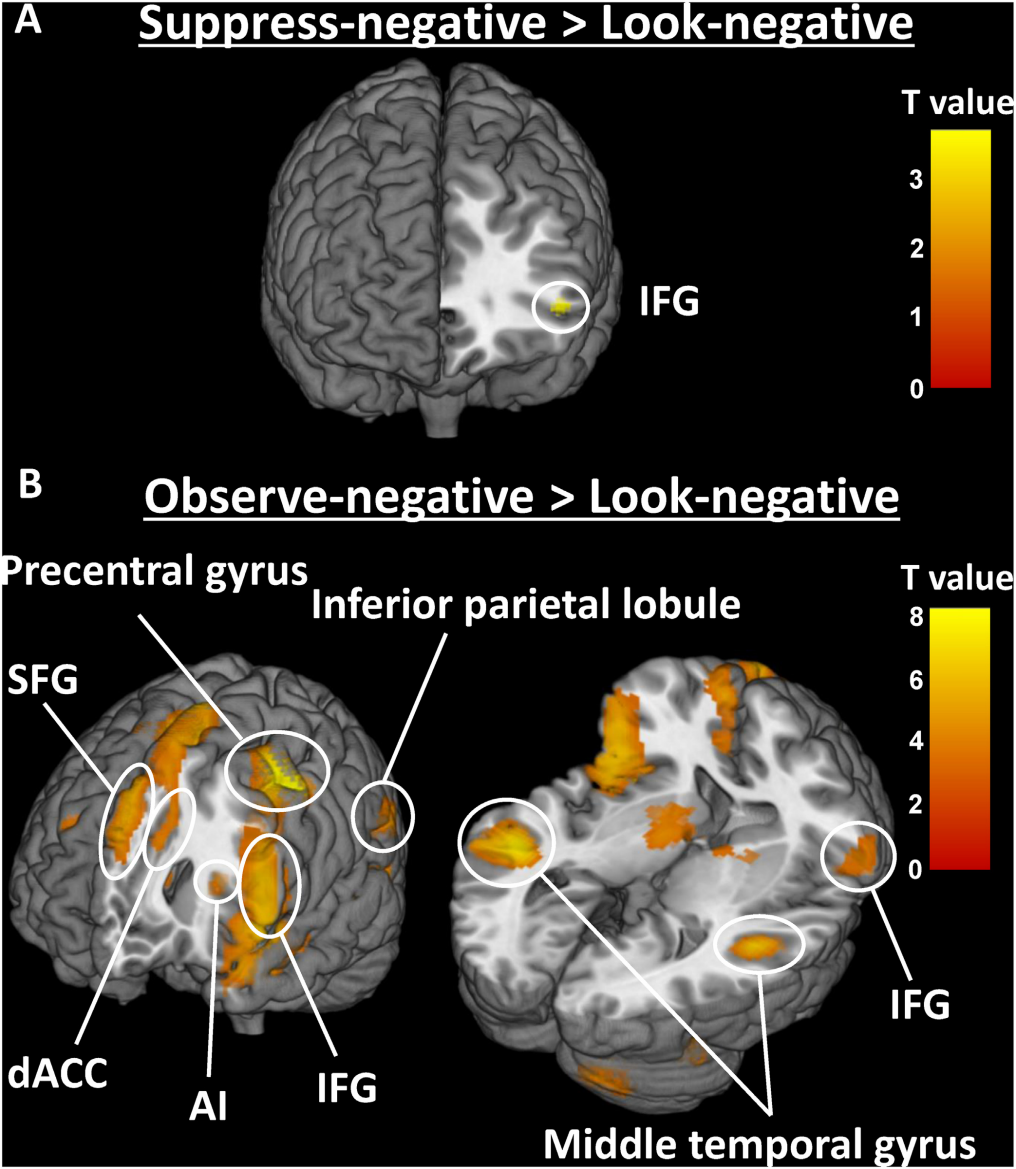
Neural activity for Suppress-negative (A) and Observe-negative (B) contrasted with Look-negative (*p* < .001 uncorrected). Regions with significantly greater activation in Suppress-negative compared to Look-negative condition included the left inferior frontal gyrus (IFG; BA47). During Observe-negative compared to Look-negative, there was greater activation in the precentral gyrus (BA6), IFG (BA44), superior frontal gyrus (SFG; BA6/8), middle temporal gyrus (MTG; BA21/22), inferior parietal lobule (IPL; BA40), putamen, and anterior insula (AI; BA13).

To examine the effect of emotion regulation in the whole brain, we computed Suppress-negative > Look-negative (Table 1 and Fig 4A) and Observe-negative > Look-negative contrasts (Table 2 and Fig 4B). We found greater activation for Suppress-negative compared to Look-negative in the left inferior frontal gyrus (IFG; Brodmann area (BA) 47). In contrast, during Observe-negative compared to Look-negative, there was greater activation in the precentral gyrus (BA6), IFG (BA44), superior frontal gyrus (SFG; BA6/8), middle temporal gyrus (MTG; BA21/22), inferior parietal lobule (IPL; BA40), putamen, and anterior insula (AI; BA13). These results suggest completely differential neural correlates for the two coping strategies studied here, such that observational (mindful) coping seems to engage wider neural networks than does suppression.

**Table 1:** Coordinates for the brain areas activated in Suppress-negative vs. Look-negative.

Area	BA	MNI coordinates (mm)			*T*	*Z*	Cluster size, *k*
		*x*	*y*	*z*			
Suppress negative > Look negative							
Left Inferior Frontal Gyrus	45	-48	32	2	3.74	3.53	23
Right Inferior Parietal Lobule	40	64	-44	42	3.72	3.52	7
Left Inferior Parietal Lobule	40	-62	-52	34	3.52	3.34	10

Height threshold: *p* < .001 uncorrected, Extent threshold: *k* = 5 voxels. The x, y, and z coordinates by which a voxel is determined referring to medial–lateral (x: positive = right), anterior–posterior (y: positive = anterior), and superior–inferior (z: positive = superior) positions denote the peak location on the MNI template. T-scores denote the difference between the two sample means compared with the dispersion and sample sizes of the two samples.Z-scores are the numbers from the unit normal distribution that give the same p value as the t statistic. Abbreviations: BA = Brodmann area; MNI = Montreal Neurological Institute template.

**Table 2:** Coordinates of the brain areas activated in Observe-negative vs. Look-negative.

Area	BA	MNI coordinates (mm)			*T*	*Z*	Cluster size, *k*
		*x*	*y*	*z*			
Observe negative > Look negative							
Left Precentral Gyrus	6	-50	0	50	8.25	6.72	3980
Left Superior Temporal Gyrus	38	-52	16	-6	6.58	5.69	
Left Inferior Frontal Gyrus	44	-52	16	16	6.22	5.44	
Left Superior Frontal Gyrus	6	-4	6	64	7.99	6.57	2802
Left Superior Frontal Gyrus	8	-10	52	46	5.74	5.11	
Left Superior Frontal Gyrus	8	-6	46	52	5.57	4.98	
Left Middle Temporal Gyrus	22	-52	-36	2	7.51	6.28	1958
Left Inferior Parietal Lobule	40	-50	-48	22	5.35	4.82	
Right Middle Temporal Gyrus	21	50	-32	0	6.63	5.72	334
Left Lentiform Nucleus		-18	8	10	5.84	5.18	751
Left Lentiform Nucleus		-12	-2	2	4.56	4.21	
Left Insula	13	-32	16	12	4.53	4.18	
Right Cerebellum		30	-68	-24	5.79	5.14	1777
Right Cerebellum		38	-62	-28	5.54	4.96	
Right Cerebellum		24	-66	-50	5.24	4.73	
Right Cingulate Gyrus	32	12	16	42	4.49	4.15	63
Right Inferior Frontal Gyrus	45	54	24	6	4.04	3.78	141
Right Inferior Frontal Gyrus	45	58	24	14	3.92	3.68	
Right Lentiform Nucleus		20	8	10	3.98	3.73	94
Right Caudate		8	2	2	3.77	3.56	
Right Superior Frontal Gyrus	9	18	54	34	3.94	3.7	74
Left Cerebellum		-32	-60	-22	3.44	3.28	16

Height threshold: *p* < .001 uncorrected, Extent threshold: *k* = 5 voxels. The x, y, and z coordinates by which a voxel is determined referring to medial–lateral (x: positive = right), anterior–posterior (y: positive = anterior), and superior–inferior (z: positive = superior) positions denote the peak location on the MNI template. T-scores denote the difference between the two sample means compared with the dispersion and sample sizes of the two samples. Z-scores are the numbers from the unit normal distribution that give the same p value as the t statistic. Abbreviations: BA = Brodmann area; MNI = Montreal Neurological Institute template.

### Functional connectivity with the AMG

**Fig 5:**
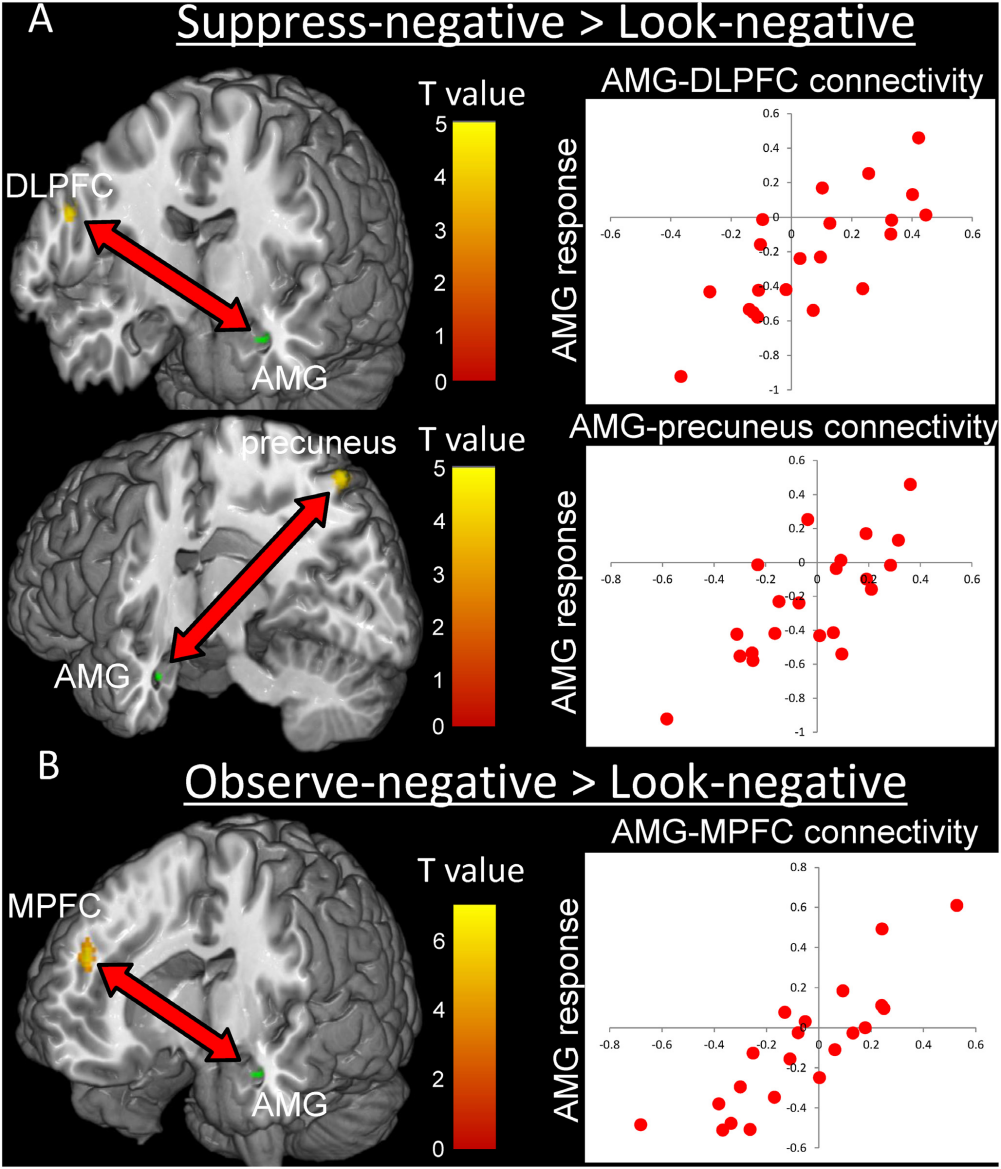
Correlation between regional AMG activity and functional connectivity to the AMG. The figures show brain regions that have more negative functional connectivity with the AMG when AMG responses were reduced during Suppress-negative (A) and Observe-negative (B) contrasted with Look-negative (*p* < .001 uncorrected).

AMG activity differences across individuals should be produced by individual differences in functional connectivity from remote regions to the AMG. In addition, the relationship between AMG activity and functional connectivity should depend on coping strategies. As expected, we found that during the Observe-negative condition (compared to Look-negative), individual AMG-MPFC connectivities were most correlated with left AMG activity across individuals, in a positive direction (*r*(19) = .87, *p* = 4×10^-7^, Table 3 and Fig 5(B)). As noted previously, the AMG has negative functional connectivity to the MPFC, and the present results mean that stronger negative functional connectivity accompanies more attenuated AMG activity (and vice versa; i.e., stronger positive functional connectivity accompanies stronger AMG activity). On the other hand, during the Suppress-negative task (contrasted with the Look-negative task), individual left AMG activity was positively correlated with multiple connectivities to remote regions (see Table 4). These include AMG-DLPFC and AMG-precuneus connectivity (*r*(19) = .76, *p* = .00007 and *r*(19) = .75, *p* = .0001, respectively; Fig 5(A)). These results indicate that functional connectivity to the AMG correlates with AMG activity in ways that differ between suppressive and observational coping: AMG activity was attenuated via negative MPFC-AMG connectivity for the observational strategy, but via negative DLPFC-AMG and precuneus-AMG connectivity for the suppression strategy.

**Table 3:** Coordinates of the brain areas activated in Observe-negative vs. Look-negative.

Area	BA	MNI coordinates (mm)			*T*	*Z*	Cluster size, *k*
		*x*	*y*	*z*			
Observe negative > Look negative							
Right Superior Frontal Gyrus	9	12	48	28	7.52	5.06	135
Right Medial Frontal Gyrus	9	6	46	36	4.61	3.73	
Right Uncus	20	32	-14	-30	5.97	4.43	21
Left Superior Frontal Gyrus	8	-12	36	50	5.33	4.12	34
Left Superior Frontal Gyrus	8	-8	40	58	4	3.37	
Left Insula	13	-38	-6	16	5.02	3.95	22
Right Superior Temporal Gyrus	41	40	-40	12	4.76	3.81	11
Right Parahippocampal Gyrus	35	24	-24	-26	4.52	3.68	11
Right Inferior Frontal Gyrus	45	48	16	22	4.19	3.48	14

Height threshold: *p* < .001 uncorrected, Extent threshold: *k* = 5 voxels. The x, y, and z coordinates by which a voxel is determined referring to medial–lateral (x: positive = right), anterior–posterior (y: positive = anterior), and superior–inferior (z: positive = superior) positions denote the peak location on the MNI template. T-scores denote the difference between the two sample means compared with the dispersion and sample sizes of the two samples. Z-scores are the numbers from the unit normal distribution that give the same p value as the t statistic. Abbreviations: BA = Brodmann area; MNI = Montreal Neurological Institute template.

**Table 4:** Coordinates of the brain areas whose connectivity with the amygdala was positively correlated with regional amygdala activity during Suppress-negative vs. Look-negative.

Area	BA	MNI coordinates (mm)			*T*	*Z*	Cluster size, *k*
		*x*	*y*	*z*			
Suppress negative > Look negative							
Left Superior Temporal Gyrus	22	-48	-10	-6	5.14	4.02	15
Left Caudate		0	12	18	5.13	4.01	23
Right Middle Frontal Gyrus	9	50	10	28	5.06	3.98	60
Right Postcentral Gyrus	5	44	-44	64	4.89	3.88	20
Right Superior Parietal Lobule	7	14	-70	66	4.87	3.88	50
Left Thalamus		-2	-8	2	4.78	3.83	14
Left Postcentral Gyrus	3	-44	-28	56	4.77	3.82	10
Right Fusiform Gyrus	19	46	-68	-20	4.7	3.78	17
Right Cingulate Gyrus	32	14	10	38	4.63	3.74	7
Left Precentral Gyrus	6	-42	-10	42	4.61	3.73	22
Right Precuneus	31	18	-56	34	4.49	3.66	10
Left Middle Temporal Gyrus	39	-50	-80	22	4.48	3.65	11
Right Middle Frontal Gyrus	6	24	-8	64	4.32	3.56	14
Left Cingulate Gyrus	24	-18	-2	46	4.11	3.43	10
Left Posterior Cingulate	31	-6	-60	24	4.03	3.39	6
Left Middle Frontal Gyrus	6	-18	-4	66	3.96	3.34	5
Right Superior Parietal Lobule	7	22	-56	62	3.92	3.32	8

Height threshold: *p* < .001 uncorrected, Extent threshold: *k* = 5 voxels. The x, y, and z coordinates by which a voxel is determined referring to medial–lateral (x: positive = right), anterior–posterior (y: positive = anterior), and superior–inferior (z: positive = superior) positions denote the peak location on the MNI template. T-scores denote the difference between the two sample means compared with the dispersion and sample sizes of the two samples. Z-scores are the numbers from the unit normal distribution that give the same p value as the t statistic. Abbreviations: BA = Brodmann area; MNI = Montreal Neurological Institute template.

## Discussion

The purpose of the present study was to examine the different neural systems underlying mindful and suppression-based emotion regulation. Subjective negative affect scores showed that both emotion regulation strategies were effective in regulating negative emotion in response to negative stimuli, consistent with evidence from previous studies of emotion suppression [29] and mindfulness [4–6]. There were corresponding decreases in AMG responses for both emotion regulation strategies. These results showed that both emotion regulation strategies were effective to regulate emotional neural responses as well as subjective emotion in response to negative stimuli.

The suppression strategy in our study activated the ventrolateral prefrontal cortex (VLPFC), which is in line with evidence that the VLPFC plays a primary role in suppressing emotion [27,29,30]. In contrast, a broader range of brain areas were deployed during the Observe (mindful) condition. The superior frontal gyrus is engaged in ‘detachment’ during emotion regulation, as reported in previous studies [3,41]. The Observe strategy used in our study involves taking an objective perspective regarding one’s own internal states. The participants therefore distanced themselves from their own states. Additionally, the dACC was activated during the Observe condition. The dACC is associated with attention [42] and the ability to accurately detect emotional signals [43], verifying that the participants in our study attended to the presented pictures instead of avoiding them, with a corresponding increased awareness of their emotional experience. The activation of the anterior insula (AI) also suggests that the participants mindfully experienced their emotional states without avoidance in the Observe condition, considering that the AI processes interoceptive information important for emotional awareness [44,45], with its structural development closely connected to mindfulness [46–48]. In sum, the Observe strategy seems to recruit neural networks involved in multiple cognitive/emotional processes, including detachment, attention, and emotional awareness.

In the Observe condition, participants were also instructed to describe their own states of mind using their inner voice. This may account for why we found activation in language areas such as the left inferior frontal gyrus, including Broca’s area and the bilateral middle temporal gyrus [49–51]. Mindfulness involves verbal labeling as a useful tool for self-awareness [19]. The present results suggest that the labeling play an important role in the regulation of emotion during use of an observational coping strategy.

Functional connectivity analyses were conducted to investigate the different neural systems underlying the two types of emotion regulation strategy. Our results revealed that largely different functional connectivity modulates AMG activity during use of the two strategies. Note that the DLPFC and the precuneus contributed to regulation of amygdala responses via functional connectivity in the Suppression condition. Reportedly, the DLPFC is activated during the tasks requiring cognitive effort [30], which leads to enhancement of sympathetic nervous activity [52]. This is consistent with evidence from previous emotion suppression studies, which showed increased peripheral responses during suppression [1,14,29]. Additionally, the precuneus underlies the default mode network in the resting brain, the activation of which is attenuated during cognitive task performance [53]. One possibility is that when participants try to suppress their negative emotional responses, they might avoid processing external stimuli. This notion is supported by a study which investigated the association between precuneus activation and visual avoidance as measured by eye tracking while spider phobic participants were presented pictures of spiders [54].

On the other hand, the MPFC contributed to regulation of amygdala responses in the Observe condition. In most cases, the amygdala has negative functional connectivity with the MPFC (e.g., [23,24,26,35]). In animal studies, electrical stimulation of the MPFC decreases the excitability of the central nucleus of the amygdala [55], and the MPFC is reported to contribute to extinction learning in human studies [26]. One possible presumption here is that the mechanism of emotion regulation in mindfulness may include extinction learning. Additionally, in the mindful coping strategy, the participants accept their emotions instead of requiring the cognitive effort represented by activation in the DLPFC. Moreover, the mindful coping strategy seemed to activate the parasympathetic nervous system [17], which is known to be modulated by MPFC activity [56]. Therefore, the mindful coping strategy in the present study might be associated with parasympathetic activity as well as regulating AMG responses via the MPFC. These neural mechanisms may promote therapeutic effects on psychopathology [9,12]. Future studies should simultaneously measure peripheral responses and brain activity during mindfulness.

The present study is constrained by one limitation. Although the Observe strategy was effective for regulating both emotional neural responses and subjective emotions in response to negative stimuli, the statistical power of the effect was low. Therefore, it is suggested that this relationship should be further examined in future studies using a larger sample of participants.

To our knowledge, this is the first time that the two types of emotion regulation—mindfulness and suppression—were compared in a single study. Both strategies attenuated AMG responses, to some extent, to emotional triggers but the regulation pathways were different: The mindful approach regulates AMG responding via connectivity from the MPFC, which is an important region for emotional awareness and mindfulness, while suppression uses functional connectivity with other regions including the DLPFC and precuneus, which should be involved in more top-down regulatory processing and which therefore requires more cognitive effort.

IAPS identification numbers for the pictures used. Neutral pictures: 1670, 2190, 2393, 2570, 5530, 7004, 7006, 7010, 7020, 7035, 7041, 7050, 7100, 7179, 7205, 7217, 7224, 7491, 7705, 7950, 9360. Negative pictures set 1: 1022, 1052, 1114, 1220, 1274, 1310, 1932, 3100, 3120, 3550, 6243, 6260, 6360, 6560, 6831, 7380, 8485, 9102, 9250, 9320, 9594. set 2: 1051, 1113, 1270, 1300, 1930, 2120, 3071, 3110, 3500, 6230, 6250, 6315, 6530, 6571, 7361, 8480, 9140, 9301, 9400, 9440, 9635.1. set 3: 1050, 1070, 1120, 1200 1230, 1275, 1525, 2095, 2220, 3030, 3101, 3130, 6212, 6244, 6300, 6510, 6570, 7359, 8230, 9300, 9630.

## Supporting Information

S1 Table Coordinates for the brain areas activated in Look-negative vs. Look-neutral. (DOC)
