# Supplementary material for: Neural Networks for Mindfulness and Emotion Suppression
Source: PLoS One. 2015 Jun 17;10(6):e0128005. doi: 10.1371/journal.pone.0128005 (PMC4471202; doi:10.1371/journal.pone.0128005)
Supplement: S1 Table — (DOC) [file pone.0128005.s001.doc]

S1 Table

| Coordinates for the brain areas activated in Look-negative vs. Look-neutral | | | | | | | | |
| --- | --- | --- | --- | --- | --- | --- | --- | --- |
| Area | | BA | MNI coordinates (mm) | | | *T* | *Z* | Cluster size, *k* |
|  |  |  | *x* | *y* | *z* |  |  |  |
|  | |  |  |  |  |  |  |  |
| Look negative > Look neutral | |  |  |  |  |  |  |  |
|  | Left Middle Occipital Gyrus | 19 | −46 | −80 | 0 | 10.13 | 7.7 | 5651 |
|  | Left Middle Occipital Gyrus | 18 | −30 | −96 | 0 | 7.46 | 6.25 |  |
|  | Left Middle Occipital Gyrus | 18 | −22 | −98 | −2 | 7.17 | 6.07 |  |
|  | Right Inferior Occipital Gyrus | 19 | 44 | −78 | −4 | 10.06 | 7.67 | 5259 |
|  | Right Middle Occipital Gyrus | 18 | 22 | −92 | −2 | 7.03 | 5.98 |  |
|  | Right Cuneus | 18 | 18 | −102 | 8 | 6.84 | 5.86 |  |
|  | Right Inferior Frontal Gyrus | 9 | 42 | 4 | 30 | 5.27 | 4.76 | 597 |
|  | Right Middle Frontal Gyrus | 6 | 48 | 8 | 56 | 4.74 | 4.35 |  |
|  | Right Superior Frontal Gyrus | 8 | 42 | 16 | 58 | 4.35 | 4.04 |  |
|  | Left Middle Frontal Gyrus | 6 | −30 | 0 | 50 | 5.11 | 4.64 | 424 |
|  | Left Middle Frontal Gyrus | 6 | −42 | 2 | 60 | 5 | 4.55 |  |
|  | Left Middle Frontal Gyrus | 6 | −52 | 4 | 48 | 3.94 | 3.7 |  |
|  | Left Superior Parietal Lobule | 7 | −12 | −68 | 56 | 4.86 | 4.44 | 717 |
|  | Left Superior Parietal Lobule | 7 | −22 | −64 | 48 | 4.62 | 4.26 |  |
|  | Right Superior Frontal Gyrus | 9 | 8 | 58 | 42 | 4.84 | 4.43 | 114 |
|  | Left Superior Frontal Gyrus | 9 | −8 | 60 | 38 | 3.78 | 3.56 |  |
|  | Right Inferior Frontal Gyrus | 47 | 46 | 28 | −6 | 4.32 | 4.02 | 198 |
|  | Right Inferior Frontal Gyrus | 47 | 38 | 26 | −6 | 4.1 | 3.83 |  |
|  | Right Inferior Frontal Gyrus | 47 | 28 | 26 | −16 | 3.95 | 3.71 |  |
|  | Right Inferior Parietal Lobule | 40 | 34 | −58 | 46 | 4.23 | 3.94 | 246 |
|  | Left Cingulate Gyrus | 32 | −8 | 22 | 44 | 4.05 | 3.79 | 215 |
|  | Right Medial Frontal Gyrus | 8 | 4 | 18 | 48 | 4.05 | 3.79 |  |
|  | Left Medial Frontal Gyrus | 8 | −8 | 14 | 52 | 3.9 | 3.66 |  |
|  | Left Thalamus |  | −22 | −28 | 0 | 4 | 3.75 | 24 |
|  | Right Superior Frontal Gyrus | 6 | 12 | 14 | 72 | 3.87 | 3.65 | 16 |
|  | Left Cuneus | 19 | −26 | −92 | 34 | 3.85 | 3.62 | 26 |
|  | Right Cerebellum |  | 32 | −54 | −42 | 3.82 | 3.6 | 20 |
|  | Right Middle Frontal Gyrus | 46 | 54 | 34 | 24 | 3.78 | 3.57 | 179 |
|  | Right Inferior Frontal Gyrus | 46 | 56 | 36 | 8 | 3.68 | 3.48 |  |
|  | Right Middle Frontal Gyrus | 46 | 52 | 26 | 26 | 3.67 | 3.47 |  |
|  | Left Superior Frontal Gyrus | 8 | −12 | 40 | 58 | 3.72 | 3.52 | 5 |
|  | Left Parahippocampal Gyrus |  | −34 | −8 | −22 | 3.71 | 3.51 | 9 |
|  | Right Middle Frontal Gyrus | 6 | 32 | 0 | 50 | 3.71 | 3.51 | 21 |
|  | Left Precuneus | 7 | −26 | −72 | 28 | 3.6 | 3.41 | 13 |
|  | Left Inferior Semi-Lunar Lobule |  | −30 | −68 | −50 | 3.58 | 3.39 | 21 |
|  | Left Inferior Frontal Gyrus | 47 | −24 | 28 | −10 | 3.56 | 3.38 | 8 |
|  | Right Cerebellar Tonsil |  | 20 | −36 | −50 | 3.52 | 3.34 | 11 |
|  | Left Middle Frontal Gyrus | 10 | −34 | 60 | 16 | 3.49 | 3.31 | 15 |
|  | Left Brainstem |  | −4 | −16 | −14 | 3.48 | 3.31 | 9 |
|  | Left Cerebellum |  | −18 | −60 | −36 | 3.48 | 3.3 | 11 |
|  | Left Precuneus | 7 | −20 | −86 | 46 | 3.46 | 3.29 | 8 |

Height threshold: *p* < .001 uncorrected, Extent threshold: *k* = 5 voxels.The x, y, and z coordinates by which a voxel is determined referring to medial –lateral (x: positive = right), anterior– posterior (y: positive = anterior), and superior– inferior (z: positive = superior) positions denote the peak location on the MNI template.

T-scores denote the difference between the two sample means compared with the dispersion and sample sizes of the two samples.

Z-scores are the numbers from the unit normal distribution that give the same p value as the t statistic.

Abbreviations: BA = Brodmann area; MNI = Montreal Neurological Institute template.
